# Tuberculous Meningitis: Diagnosis and Treatment Overview

**DOI:** 10.1155/2011/798764

**Published:** 2011-12-21

**Authors:** Grace E. Marx, Edward D. Chan

**Affiliations:** ^1^Department of Medicine, University of Colorado Denver Anschutz Medical Campus, Aurora, CO 80045, USA; ^2^Division of Pulmonary Sciences and Critical Care Medicine, University of Colorado Denver Anschutz Medical Campus, Aurora, CO 80045, USA; ^3^Denver Veterans Affairs Medical Center, Denver, CO 80220-3808, USA; ^4^Department of Medicine, National Jewish Health, Denver, CO 80206, USA; ^5^Program in Cell Biology, National Jewish Health, Denver, CO 80206, USA

## Abstract

Tuberculous meningitis (TBM) is the most common form of central nervous system tuberculosis (TB) and has very high morbidity and mortality. TBM is typically a subacute disease with symptoms that may persist for weeks before diagnosis. Characteristic cerebrospinal fluid (CSF) findings of TBM include a lymphocytic-predominant pleiocytosis, elevated protein, and low glucose. CSF acid-fast smear and culture have relatively low sensitivity but yield is increased with multiple, large volume samples. Nucleic acid amplification of the CSF by PCR is highly specific but suboptimal sensitivity precludes ruling out TBM with a negative test. Treatment for TBM should be initiated as soon as clinical suspicion is supported by initial CSF studies. Empiric treatment should include at least four first-line drugs, preferably isoniazid, rifampin, pyrazinamide, and streptomycin or ethambutol; the role of fluoroquinolones remains to be determined. Adjunctive treatment with corticosteroids has been shown to improve mortality with TBM. In HIV-positive individuals with TBM, important treatment considerations include drug interactions, development of immune reconstitution inflammatory syndrome, unclear benefit of adjunctive corticosteroids, and higher rates of drug-resistant TB. Testing the efficacy of second-line and new anti-TB drugs in animal models of experimental TBM is needed to help determine the optimal regimen for drug-resistant TB.

## 1. Introduction

Tuberculous meningitis (TBM) is caused by *Mycobacterium tuberculosis* (*M. tuberculosis*) and is the most common form of central nervous system (CNS) tuberculosis (TB). TBM is associated with a high frequency of neurologic sequelae and mortality if not treated promptly [1–5]. TBM is rare in developed countries with about 100 to 150 cases occurring annually in the US, less than 3% of the estimated 4,100 annual cases of bacterial meningitis [6, 7]. The disease occurs when subependymal or subpial tubercles, also known as “Rich foci” seeded during bacillemia of primary infection or disseminated disease, rupture into the subarachnoid space [8]. Individuals with increased risk for TBM include young children with primary TB and patients with immunodeficiency caused by aging, malnutrition, or disorders such as HIV and cancer [9, 10]. The use of antitumor necrosis factor-alpha (TNF*α*) neutralizing antibody has also been associated with increased risk of extrapulmonary TB including TBM [11]. Most have no known history of TB, but evidence of extrameningeal disease (e.g., pulmonary) can be found in about half of patients [3, 4]. The tuberculin skin test is positive in only about 50% of patients with TBM. In low TB prevalence areas, TBM is most commonly seen with reactivation TB.

## 2. Objective and Method

The goal of this overview is to describe evidence-based diagnostic and treatment approaches of TBM. This paper was written for clinicians seeking a practical summary of this topic. While this paper focuses on these aspects of TBM, a brief overview of the clinical manifestations of TBM as well as past and current animal models of TBM treatment will be discussed.

Literature in this field was systematically identified on PubMed using the key words “tuberculous meningitis,” “tuberculosis cerebrospinal fluid,” and “tuberculosis nervous system,” as well as combing through the bibliography of relevant papers. More recent articles describing new findings in the field were given particular attention.

## 3. Clinical Manifestations

TBM is typically a subacute disease. In one seminal review, symptoms were present for a median of 10 days (range, one day to nine months) prior to diagnosis [4]. A prodromal phase of low-grade fever, malaise, headache, dizziness, vomiting, and/or personality changes may persist for a few weeks, after which patients can then develop more severe headache, altered mental status, stroke, hydrocephalus, and cranial neuropathies. Seizures are uncommon manifestations of TBM in adults and when present should prompt the clinician to consider alternate diagnoses such as bacterial or viral meningitis or cerebral tuberculoma; in contrast, seizures are commonly seen in children with TBM, occurring in up to 50% of pediatric cases [12]. The clinical features of TBM are the result of basilar meningeal fibrosis and vascular inflammation [13]. Classic features of bacterial meningitis, such as stiff neck and fever, may be absent. When allowed to progress without treatment, coma and death almost always ensue. In survivors of TBM, neurologic sequelae may occur that include mental retardation in children, sensorineural hearing loss, hydrocephalus, cranial nerve palsies, stroke-associated lateralizing neurological deficits, seizures, and coma [14].

## 4. Diagnosis

The diagnosis of TBM can be difficult and may be based only on clinical and preliminary cerebrospinal fluid (CSF) findings without definitive microbiologic confirmation. Certain clinical characteristics such as longer duration of symptoms (>six days), moderate CSF pleiocytosis, and the presence of focal deficits increase the probability of TBM [15, 16]. Characteristic CSF findings of TBM include the following:

- lymphocytic-predominant pleiocytosis. Total white cell counts are usually between 100 and 500 cells/*μ*L. Very early in the disease, lower counts and neutrophil predominance may be present,
- elevated protein levels, typically between 100 and 500 mg/dL,
- low glucose, usually less than 45 mg/dL or CSF: plasma ratio <0.5.

CSF sample should be sent for acid-fast smear with the important caveat that a single sample has low sensitivity, on the order of 20%–40% [17]. Several daily large volume (10–15 mL) lumbar punctures are often needed for a microbiologic diagnosis; sensitivity increases to >85% when four spinal taps are performed [18]. Early studies demonstrated that acid-fast stains can detect up to 80% [18] although results are highly dependent on CSF volume, timeliness of sample delivery to the lab and analysis, and the technical expertise of lab personnel. While culture can take several weeks and also has low sensitivity (~40–80%), it should be performed to determine drug susceptibility. Drug-resistant strains have important prognostic and treatment implications; indeed, TBM due to isoniazid- (INH-) resistant *M. tuberculosis* strains have been associated with a twofold increase in mortality [19].

Given the relatively low sensitivity of acid-fast smear and inherent delay in culture, newer diagnostic methods for TBM have been more recently developed [17]. Although ELISA assays have been developed to detect antibodies directed against specific mycobacterial antigens in the CSF with varying sensitivities, their limited availability precludes their use as point-of-care tests in resource-poor countries [17, 20]. A recent study in children aged 6–24 months suggests that a CSF adenosine deaminase level of ≥10 U/L has >90% sensitivity and specificity of diagnosing TBM [21]. However, other studies have shown poor specificity of adenosine deaminase for TBM in certain populations, particularly in HIV-infected adults with concurrent infections or cerebral lymphomas [22].

Comparison of microscopy/culture of large CSF volumes to nucleic acid amplification (NAA) has shown that sensitivity of these methods for the diagnosis of TBM is similar [23]. A meta-analysis determined that commercial NAA assays utilizing polymerase chain reaction (PCR) for the diagnosis of TBM had an overall sensitivity of 56% and a specificity of 98% [24]. The surprisingly poor sensitivity is likely due to the fact that most PCR-based studies use a single target for amplification which can result in false-negative results due to the absence of the target gene in some TB isolates [25]. Newer PCR tests amplify several target genes simultaneously and have been shown to result in much higher sensitivities in the range of 85%–95% [26]. Currently, most experts conclude that commercial NAA tests can confirm TBM but cannot rule it out [27]. Thus, it bears emphasizing that a negative CSF examination for acid-fast bacilli or *M. tuberculosis* DNA neither excludes the diagnosis of TBM nor obviates the need for empiric therapy if the clinical suspicion is high. After starting treatment, the sensitivity of CSF smear and culture decreases rapidly, while mycobacterial DNA may be detectable in the CSF for up to a month after treatment initiation [28].

Diagnosis of TBM can be helped by neuroimaging. Classic neuroradiologic features of TBM are basal meningeal enhancement and hydrocephalus [17]. Hypodensities due to cerebral infarcts, cerebral edema, and nodular enhancing lesions may also be seen. Magnetic resonance imaging (MRI) is the imaging test of choice for visualizing abnormalities associated with TBM, as it is superior to computed tomography (CT) for evaluating the brainstem and spine. The T2-weighted MRI imaging has been shown to be particularly good at demonstrating brainstem pathology; diffusion-weighted imaging (DWI) is best at detection of acute cerebral infarcts due to TBM [29]. However, CT is adequate for urgent evaluation of TBM-associated hydrocephalus for possible surgical intervention.

## 5. Treatment

### 5.1. Antimicrobial Therapy

Timely treatment dramatically improves the outcome of TBM. Thus, empiric treatment is warranted when clinical features and CSF findings are suggestive of TBM even before microbiologic confirmation. The recommended treatment regimen for presumed drug-susceptible TBM consists of two months of daily INH, rifampin (RIF), pyrazinamide (PZA), and either streptomycin (SM), or ethambutol (EMB), followed by 7–10 months of INH and RIF (Table 1) [17, 30–34]. INH is considered the most critical of the first-line agents due to its excellent CSF penetration and high bactericidal activity (Table 2) [35–39]. While RIF penetrates the CSF less freely, the high mortality of TBM due to RIF-resistant strains has confirmed its importance [40]. PZA has excellent penetration into the CSF and is a key drug in reducing the total treatment time for drug-susceptible TB [41]. Hence, if PZA cannot be tolerated, the treatment course for TBM should be lengthened to a total of 18 months. While SM or EMB are traditionally used as the fourth anti-TB agent in TBM, neither penetrates the CSF well in the absence of inflammation and both can produce significant toxicity with long-term use [41]. It bears emphasizing that not only the choice of antimicrobials, but also the dose used and duration of treatment are empiric in TBM and largely based on the treatment of pulmonary TB.

Given that the newer generation fluoroquinolones (FQN), for example, levofloxacin and moxifloxacin, have strong activity against most strains of *M. tuberculosis* and have excellent CSF penetration and safety profiles, FQN would appear to have great potential as part of first-line therapy for TBM. In a randomized controlled study for TBM treatment, addition of an FQN to standard regimen enhanced anti-TB performance as measured by various clinical parameters. Although there was no significant difference in mortality, the study was likely not adequately powered to demonstrate such an effect [38]. It is important to note that serum FQN concentrations are lowered by concurrent RIF use; furthermore, the optimal area-under-the-curve to minimum inhibitory concentration ratio for FQN as anti-TB agents has not been well described. Another randomized controlled study is currently underway to evaluate treatment of TBM with high-dose RIF and levofloxacin compared to standard treatment [42]; if they have positive results, the recommended standard treatment may change in the near future.

No controlled trials have been published to date for the treatment of multidrug resistant (MDR) TBM, defined as resistance to at least INH and RIF. Furthermore, very few studies have been published on the CSF penetrance of many of the second-line and newer anti-TB agents. Clinicians of patients with MDR-TBM are left to extrapolate from guidelines for the treatment of pulmonary MDR-TB. The World Health Organization recommends for pulmonary MDR-TB the use of a minimum of four agents to which the *M. tuberculosis* strain has known or suspected susceptibility including use of any first-line oral agents to which the strain remains susceptible, an injectable agent (i.e., an aminoglycoside or capreomycin), an FQN, and then adding other second-line agents as needed for a total of at least four drugs [34]. CSF penetration of the first- and second-line anti-TB drugs are shown in Table 2 [35, 43–49].

Among new anti-TB agents, bedaquiline (TMC207, a diarylquinoline) and delamanid (OPC-67683, a nitro-dihydroimidazo-oxazole) appear most promising, as they are both in phase III clinical trials [50]. Three additional novel agents, sudoterb (LL3858, a pyrrole derivative), PA-824 (a nitroimidazo-oxazine), and SQ109 (an analogue of EMB) are currently in phase II trials [50, 51]. Their ability to penetrate the CSF has yet to be adequately studied (Table 2).

### 5.2. Adjunctive Corticosteroid Therapy

Much of the neurologic sequelae of TBM is considered to be due to an overexuberant host-inflammatory response that causes tissue injury and brain edema [52]. Since the middle of the 20th century, systemic corticosteroids have been used as adjunctive treatment for TBM on the basis of the notion that dampening of the inflammatory response can lessen morbidity and mortality, a reasonable hypothesis as the brain is confined to a fixed space. Indeed, adjunctive corticosteroid treatment of pyogenic bacterial meningitis has shown efficacy in certain groups of patients [53, 54] although this is controversial [55, 56]. In attempting to determine the cell type responsible for inciting the inflammatory response, Rock et al. [2] found that *M. tuberculosis* was much more likely to infect brain tissue macrophages (microglial cells) with marked increases in production of proinflammatory cytokines and chemokines than stromal brain cells (astrocytes). In this *in vitro* study, coincubation of TB-infected microglial cells with dexamethasone significantly inhibited production of inflammatory mediators [2]. Although there has long been concern that corticosteroids may reduce CSF penetration of anti-TB drugs [13], one small study demonstrated that corticosteroids had no effect on CSF penetrance of first-line anti-TB agents [46]. A Cochrane meta-analysis of seven randomized controlled trials comprised a total of 1140 participants concluded that corticosteroids improved outcome in HIV-negative children and adults with TBM (RR 0.78) [57]. These results were strongly influenced by a study of 545 adults with TBM in Vietnam showing that treatment with dexamethasone was associated with significantly reduced mortality at nine months of followup [58]. One possible explanation for the survival benefit in the Vietnamese study is that the anti-inflammatory effects of corticosteroids reduced the number of severe adverse events (9.5% versus 16%), particularly hepatitis, preventing the interruption of the first-line anti-TB drug regimen [58].

Since there are no controlled trials comparing corticosteroid regimens, treatment choice should be based on those found to be effective in published trials. One recommended regimen for children is dexamethasone 12 mg/day IM (8 mg/day for children weighing ≤25 kg) for three weeks, followed by gradual taper over the next three weeks [59]. In the large study in Vietnam, patients with mild disease received intravenous dexamethasone 0.3 mg/kg/day × 1 week, 0.2 mg/kg/day × 1 week, and then four weeks of tapering oral therapy [58]. For patients with more severe TBM, intravenous dexamethasone was given for four weeks (1 week each of 0.4 mg/kg/day, 0.3 mg/kg/day, 0.2 mg/kg/day, and 0.1 mg/kg/day), followed by four weeks of tapering oral dexamethasone therapy [58].

While neutralization of TNF*α* predisposes individuals to TB including TBM [11], TNF*α* is also considered to play an important role in contributing to the pathogenesis of TBM [60–63], consistent with the aforementioned deleterious effects of the CNS inflammatory response. Indeed, Tsenova et al. showed that the addition of thalidomide, a potent inhibitor of TNF*α*, to antibiotics was superior to antibiotics alone in protecting rabbits from dying (50% reduction in mortality) in their model of TBM [62]. In addition, there was marked reduction in TNF*α* levels in both CSF and blood as well as a decrease in leukocytosis and brain pathology in rabbits that received thalidomide [62].

### 5.3. Fluid Management in TBM

In patients with TBM, there may be nonosmotic stimuli for antidiuretic hormone (ADH) expression, resulting in a syndrome of inappropriate ADH (SIADH) release. While ADH itself may not aggravate cerebral edema, acute development of significant hyposmotic hyponatremia may worsen cerebral edema due to water shifting from the intravascular compartment into the extravascular (intracellular and extracellular) space of the brain. While restriction of water intake is a mainstay of SIADH treatment, hypovolemia should be avoided, since it may decrease cerebral perfusion as well as serve as a stimulus for further ADH release. In a comprehensive review of this issue, it was noted that fluid restriction to prevent cerebral edema in TBM is unjustified [64]. Instead, it was recommended that a euvolemic state should be the goal to maintain cerebral perfusion as well as to prevent hypovolemia-induced ADH release. If symptomatic, acute hyponatremia does not respond to anti-TB treatment and appropriate fluid restriction (while maintaining euvolemia), use of V2 (ADH) receptor antagonist should be considered although, to the best of our knowledge, this has not been studied in TBM. Care must be taken, however, to prevent too rapid of correction of chronic hyponatremia due to the risk of precipitating osmotic demyelination syndrome.

### 5.4. Surgical Intervention in TBM Hydrocephalus

Hydrocephalus is a common complication of TBM; prevalence has been documented in >75% of patients in several published series [65, 66]. Ventriculoperitoneal shunt placement and endoscopic third ventriculostomy are surgical techniques which have been demonstrated to relieve elevated intracranial pressure (ICP) in TBM, leading to improved neurological outcomes [67, 68]. Children are at particularly high risk for hydrocephalus and elevated ICP. In a study of 217 children with TBM in South Africa, 30% required ventriculoperitoneal shunting for either noncommunicating hydrocephalus or failure of medical therapy with diuretics in communicating hydrocephalus [69]. Historically, surgical intervention was only recommended with grade 2 or 3 TBM hydrocephalus (normal or mildly altered sensorium; easily arousable) due to increased mortality and risk of poor surgical outcome in patients with grade 4 disease (deeply comatose). However, a retrospective analysis of 95 patients with grade 4-associated hydrocephalus who underwent shunt placement demonstrated favorable outcomes in 33%–45% of patients, suggesting that there may be a role for surgical intervention even in advanced TBM hydrocephalus [70]. In this study, poor neurological outcomes after shunt placement were associated with age < three years and > three days in duration of symptoms.

### 5.5. Treatment Issues of TBM in Patients with Concurrent HIV Infection

TB is the most common opportunistic infection in HIV-infected persons, and HIV infection is an independent risk factor for extrapulmonary TB including meningitis [71]. For these reasons, diagnosis of TBM should automatically trigger testing for HIV infection. In general, the diagnosis and treatment of TBM in HIV-infected individuals is similar in principle to non-HIV infected subjects although there are a few notable caveats, including the potential development of immune reconstitution inflammatory syndrome (IRIS), drug interactions and toxicities with concomitant anti-TB and antiretroviral (ARV) therapy, questionable efficacy of adjunctive corticosteroids, and higher prevalence of drug-resistant TB in HIV-positive populations.

Treatment of HIV with ARV therapy can result in IRIS, causing clinical exacerbation of TBM. Indeed, in high HIV prevalent settings, CNS TB complicated by IRIS has been shown to be the most frequent cause for neurological deterioration in patients newly starting ARV therapy [72]. Risk factors for IRIS include a high pathogen load (e.g., miliary TB), very low CD4 T-cell count (<50 cells/*μ*L) when ARV therapy is initiated [73], and concurrent initiation of ARV and anti-TB therapy [74].

Concurrent ARV and anti-TB therapy carries the risk of drug interactions and toxicities. However, delaying ARV therapy in patients coinfected with HIV and TB has been associated with higher mortality [75]. Nevertheless, due to the possibility of IRIS with ARV initiation, most guidelines do not recommend simultaneous initiation of ARV and anti-TB medications. A recent randomized controlled trial comparing mortality in patients started on immediate ARV at the time of diagnosis of TBM and HIV versus patients started on ARV two months after diagnosis found significantly more serious adverse events in the immediate arm [74]. Mortality did not differ significantly, but there was a trend towards greater all-cause mortality in the immediate ARV group at nine months followup. The World Health Organization recommends that anti-TB therapy be started first, followed by ARV treatment within eight weeks [34]. The Center for Disease Control and Prevention recommends that for patients with CD4 counts <100 cells/*μ*L, ARV therapy be started after two weeks of anti-TB therapy [76].

The benefit of adjunctive corticosteroid treatment for TBM in patients coinfected with HIV has not been demonstrated [71]. In the large study of Vietnamese adults with TBM, no mortality benefit from dexamethasone was found in the subgroup of 98 patients who were coinfected with HIV [58]. Thus, at the present time, the benefit of adjunctive corticosteroid treatment in HIV-infected individuals remains uncertain [57] although the theoretical benefit of corticosteroids to decrease TB-associated IRIS has led some experts to prescribe them to this population.

There is also evidence that a particularly virulent strain of TB, the W-Beijing genotype, is associated with HIV infection and high levels of resistance in TBM [77]. Multiple studies have shown MDR-TB to be more commonly found in HIV-infected patients with concurrent TBM [78–80], often leading to treatment failure and very high mortality. In high HIV prevalence settings and in all HIV-infected patients, daily anti-TB treatment as directly observed therapy should be given in order to reduce relapse and treatment failure [34, 81]. It is important to note that HIV coinfection alone, even without TB drug resistance, confers worse outcomes in TBM. HIV coinfection was shown to be associated with 3.5 times higher mortality in a retrospective cohort study of TBM patients in the United States from 1993–2005 [19].

## 6. Prognosis

Prognosis of TBM largely depends on neurologic status at the time of presentation, and time-to-treatment initiation. While the course of TBM is generally not as rapid or fulminant as meningitis due to pyogenic bacteria, empiric treatment should be initiated as soon as the diagnosis is suspected as any delay in treatment can worsen outcome. Various case series indicate a mortality rate of 7%–65% in developed countries, and up to 69% in underdeveloped areas [3–5]. Mortality risk is highest in those with comorbidities, severe neurologic involvement on admission, rapid progression of disease, and advanced or very young age. Neurologic sequelae occur in up to 50% of survivors [5].

## 7. Animal Models Are Needed to Advance Our Understanding and Treatment of TBM

Animal models are critically important in testing the efficacy of new drugs and vaccines against TB [82]. The challenge of animal models of TBM is that TBM in humans is considered to typically occur a certain period of time after a primary infection through the respiratory tract, a condition that would be difficult to mimic in experimental animals. Indeed, all animal models of TBM resort to direct inoculation of *M. tuberculosis* into the CNS. The rabbit model of TBM, in which mycobacteria are inoculated directly into the cisterna magna, is perhaps the most well-established animal model of TBM [8, 62]. Therapeutic studies examining efficacy of antibiotics, vaccines, and adjunctive agents such as thalidomide in the context of TBM have been studied in the rabbit model [62, 83, 84]. While the murine model of TB is more tractable than rabbits due to the greater variety of mouse reagents available and lower cost in conducting the studies, the immunologic and clinical responses of mice to experimental TBM do not mimic as well as rabbits to human TBM [85].

Despite the fact that BCG vaccination is suboptimal in protecting against pulmonary TB [86, 87], it is considered to be relatively efficacious in protecting against childhood TBM [88]. Tsenova et al. showed in a rabbit model of TBM that while BCG provided protection against the laboratory strain *M. tuberculosis* H37Rv, it afforded significantly less protection against a hypervirulent clinical strain (W-Beijing HN878), particularly against CNS disease [84]. In BCG-vaccinated mice challenged with W-Beijing HN878, there was significantly greater infiltration of the subarachnoid space by lymphocytes and macrophages, coincident with greater bacterial burden and worse CNS pathology score [84]. An important lesson from this study is that in the search for more efficacious TB vaccines, it is important to test the vaccine in animals challenged with relevant, clinical strains of *M. tuberculosis*.

## 8. Conclusion

Meningitis is the most deadly form of TB, particularly in persons coinfected with HIV. Early diagnosis and treatment can dramatically reduce the high mortality associated with this disease. In general, treatment should be at least nine months in duration and should be comprised of at least four agents to which the *M. tuberculosis* strain has known or suspected susceptibilities. Adjunctive corticosteroid treatment should be considered, particularly in persons without concurrent HIV infection. In order to guide therapy, it is optimal to base treatment on TB resistance patterns, especially in HIV-coinfected persons who carry high risk for drug-resistant TB. More studies are needed to evaluate CSF penetration of newer TB agents to facilitate development of better treatment regimens for both drug-susceptible and drug-resistant TBM. Additionally, randomized controlled trials to optimize treatment for MDR-TBM are important to find the best possible combination of drugs available and to standardize treatment.

## Figures and Tables

**Table 1:** Recommended standard treatment regimen for drug-susceptible TBM.

Treatment phase and anti-TB agent	Recommended dose (mg/kg/day)	Maximum dose (mg/day)	Potential side effects	Duration of treatment
Isoniazid	5–10	300	hepatotoxicity peripheral neuropathy	Minimum of 9 months

Rifampin	10	450 (<50 kg) 600 (≥50 kg)	hepatotoxicity, rash, flu-like syndrome, and multiple drug interactions.	Minimum of 9 months

Pyrazinamide	25–30	1500 (<50 kg) 2000 (≥50 kg)	hepatotoxicity, arthralgia, gastrointestinal upset, anorexia, and photosensitization of the skin	2 months

Streptomycin (IM)*	15 in adults (30 in children)	1000	nephrotoxicity, ototoxicity, and vestibular toxicity	2 months

Ethambutol*	15–20	1600 in adults (1000 in HIV (−) and 2500 in HIV (+) children)	optic neuritis, peripheral neuritis, arthralgia, and gastrointestinal upset	2 months

*For empiric induction treatment for presumed drug-susceptible *M. tuberculosis*, either streptomycin or ethambutol is recommended as the fourth agent.

**Table 2:** Pharmacokinetic activity and CSF penetration of anti-TB drugs.

	Anti-TB drug	Activity	CSF penetration
1st-line drugs	Isoniazid	Cidal	90%–95%
	Rifampin	Cidal	5%–25%
	Pyrazinamide	Cidal	95%–100%
	Streptomycin	Static	20%–25%
	Ethambutol	Static	10%–50%
	Ciprofloxacin	Cidal	15%–35%
	Levofloxacin	Cidal	60%–80%
	Moxifloxacin	Cidal	70%–80%

2nd-line drugs	Ethionamide	Cidal	80%–95%
	Cycloserine	Static	40%–70%
	Amikacin	Cidal	10%–25%
	Streptomycin	Cidal	10%–20%
	Capreomycin	Static	unknown
	Para-aminosalicylic acid	Static	unknown
	Thioacetazone	Static	unknown
	Linezolid	Cidal	80%–100%

New agents	Bedaquiline (TMC207)	Cidal	unknown
	Delamanid (OPC-67683)	Cidal	unknown

Cidal: bactericidal,

Static: bacteriostatic.
